# *Trypanosoma brucei brucei* causes a rapid and persistent influx of neutrophils in the spleen of infected mice

**DOI:** 10.1111/pim.12664

**Published:** 2019-08-21

**Authors:** Violette Deleeuw, Hien Thi Thu Phạm, Isabel De Poorter, Ibo Janssens, Carl De Trez, Magdalena Radwanska, Stefan Magez

**Affiliations:** ^1^ Laboratory for Biomedical Research Ghent University Global Campus Incheon South Korea; ^2^ Laboratory for Cellular and Molecular Immunology Vrije Universiteit Brussel Brussels Belgium; ^3^ Department of Biochemistry and Microbiology Ghent University Ghent Belgium; ^4^ Department of Hematology Erasmus MC Rotterdam The Netherlands; ^5^ Laboratory for Experimental Hematology University of Antwerp Edegem Belgium; ^6^ Department of Biomedical Molecular Biology Ghent University Ghent Belgium

**Keywords:** cell‐mediated immunity, neutrophil, *Trypanosoma* spp.

## Abstract

Trypanosomosis is a chronic parasitic infection, affecting both humans and livestock. A common hallmark of experimental murine infections is the occurrence of inflammation and the associated remodelling of the spleen compartment. The latter involves the depletion of several lymphocyte populations, the induction of T‐cell‐mediated immune suppression, and the activation of monocyte/macrophage cell populations. Here, we show that in experimental *T b brucei* infections in mice, these changes are accompanied by the alteration of the spleen neutrophil compartment. Indeed, mature neutrophils are rapidly recruited to the spleen, and cell numbers remain elevated during the entire infection. Following the second peak of parasitemia, the neutrophil cell influx coincides with the rapid reduction of splenic marginal zone (MZ)B and follicular (Fo)B cells, as well as CD8^+^ T and NK1.1^+^ cells, the latter encompassing both natural killer (NK) and natural killer T (NKT) cells. This report is the first to show a comprehensive overview of all alterations in spleen cell populations, measured with short intervals throughout the entire course of an experimental *T b brucei* infection. These data provide new insights into the dynamic interlinked changes in spleen cell numbers associated with trypanosomosis‐associated immunopathology.

## 1 INTRODUCTION

*Trypanosoma brucei* is one of the causative agents of African Trypanosomosis.^1^ These parasites are continuously exposed to attacks by host antibodies, type 1 proinflammatory cytokines and nitric oxide (NO).^2^, ^3^, ^4^ In combination, these molecules can have both direct and indirect trypanotoxic activities. Prolonged inflammation is however also a detrimental hallmark of the infection for the host itself. Indeed, trypanosomosis‐associated immunopathology is linked to excessive activation of the monocyte/macrophage compartment,^5^, ^6^, ^7^, ^8^ and results in T‐cell‐mediated immune suppression 9, 10 as well as the depletion of several host lymphocyte populations.^5^, ^9^, ^11^, ^12^, ^13^ The latter has been addressed at very specific time points of infection, but so far, comprehensive data detailing with the quantitative dynamic changes of these populations throughout infection is lacking. In particular, no published information is available on systematic changes of the mature spleen neutrophil population throughout the entire course of infection covering multiple time points of the early, intermediate and late‐stage of parasitemia.

Neutrophils are known to play a key role in the first line of defence against invading pathogens via the innate arm of the immune system. Upon arrival at the site of inflammation, neutrophils engage their effector functions by eliminating invading pathogens and trigger inflammatory reactions.^14^, ^15^, ^16^ However, recent data demonstrate that neutrophils can also extend their functions beyond their role in pathogen clearance and can play a role in promoting parasite survival, in particular, during the onset of tsetse‐transmitted trypanosomosis.^17^ The lack of systematic data on quantitative changes in spleen cell numbers throughout infection prompted the data collection reported here.

## 2 MATERIAL AND METHODS

### 2.1 Parasites and infection in mice

Eight‐week‐old female C57BL/6 mice were purchased from Koatech (Gyeonggi‐do, Republic of Korea) and infected by intraperitoneal injection using 5 × 10^3^ *T b brucei* AnTat1.1E. Experiments were approved by the GUGC IACUC protocol n° LM16‐839/2018‐006. Parasitemia was assessed as previously described.^18^

### 2.2 Cell isolation and flow cytometry assay

Single‐cell spleen suspensions were prepared at 0, 4, 5, 6, 7, 8, 9, 10, 14, 17, 21, 24 and 28 days post‐infection (dpi) as previously described.^13^ Unless otherwise stated, cell suspensions were re‐suspended in 0.05% FBS BD FACSFlow Sheath Fluid. Cell washings were carried out by centrifugation at 314 g for 7 minutes. Incubations were performed at 4°C for 30 minutes. Non‐specific binding sites were blocked using anti‐CD16/CD32 (Fc γ III/II block—final dilution 1/1000). Afterwards, 5 × 10^5^ cells were incubated with antibody cocktails (dilution of 1/600), using anti‐B220‐FITC, anti‐CD1d‐PE, anti‐CD138‐PE/CY7, anti‐CD93‐APC, anti‐CD4‐FITC, anti‐CD8a‐PE, anti‐TCR β chain‐APC, anti‐Ly6G‐AlexaFluor488, anti‐Ly6C‐PE, anti‐CD11b‐APC, anti‐NK1.1‐APC and anti‐Ter119‐PE (BioLegend, San Diego, CA, USA), 1 µg of 7‐amino‐actinomycin D (7AAD) to exclude nonviable cells, and finally analysed using a BD Accuri™ C6 Plus flow cytometer.

### 2.3 Statistical analysis

Prism^®^ 7.0 software (GraphPad Software Inc) was used to graphically represent data and perform statistical analysis, using unpaired student *t* tests. Data are presented as mean ± SD.

## 3 RESULTS

**Figure 1:**
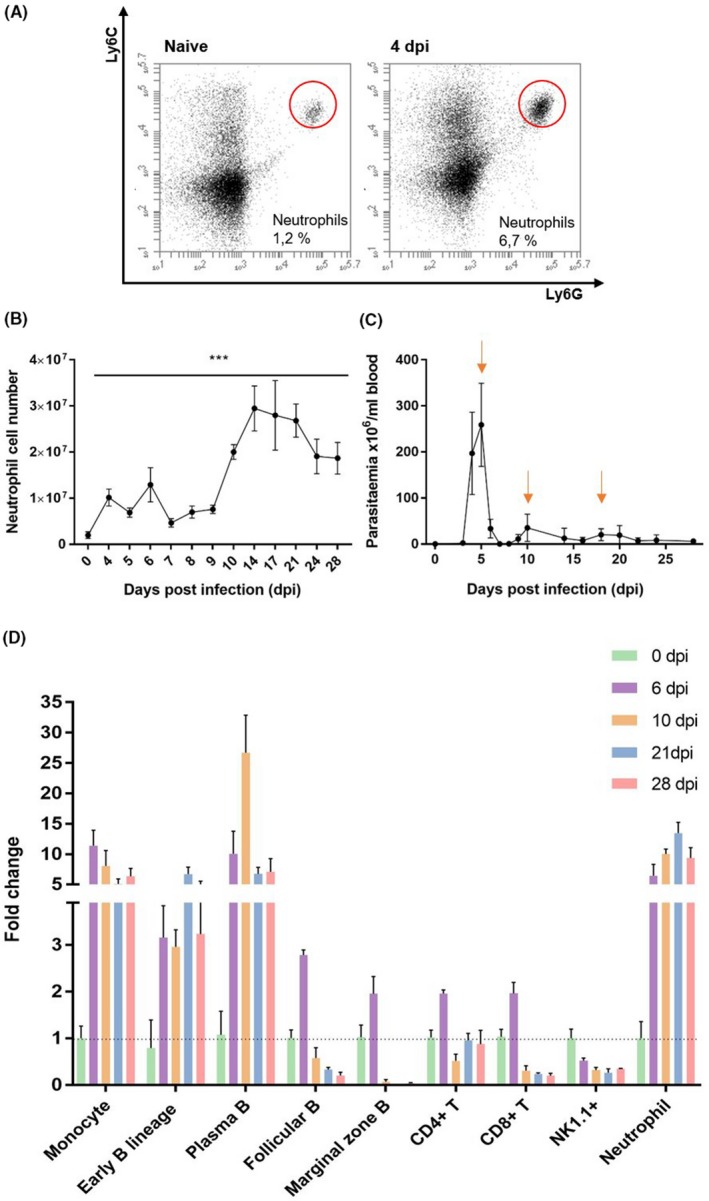
Alterations in spleen immune cell populations and parasitemia of *Trypanosoma brucei brucei* infected C57BL/6 mice. A, Flow cytometry plots of CD11b^+^Ly6C^Int^Ly6G^+^ spleen neutrophils (naive and 4 dpi, one representative result). B, Dynamic changes in spleen neutrophil numbers throughout *T b brucei* AnTat1.1E infections (n = 3 mice per time point). Statistical analysis is performed by comparing each time point to the data obtained of uninfected controls (0 dpi). ***P < .001. C) Parasitemia levels of *T b brucei* AnTat1.1E infected mice. Parasitemia peaks are indicated by arrows (n = 5 mice per time point). D) Fold change in cell number of early B lineage, plasma B, follicular (Fo)B, marginal zone (MZ)B, CD4^+^ T, CD8^+^ T, and NK1.1^+^ cells, monocytes, and neutrophils during *T b brucei* AnTat1.1E infection (n = 3 mice per time point). Absolute cell numbers were obtained by multiplying the viable spleen cell count with the percentage value obtained by flow cytometry for every specific cell population (excluding 7AAD^+^ and Ter119^+^ cells). The following criteria were used; Mature neutrophils: CD11b^+^Ly6C^Int^Ly6G^+^, Monocytes: CD11b^+^Ly6C^+^Ly6G^‐^, Early B lineage: B220^+^CD138^‐^CD93^+^, †Plasma B: B220^Int^CD138^+^, FoB: B220^+^CD138^‐^CD93^‐^CD1d^‐^, MZB: B220^+^CD138^‐^CD93^‐^CD1d^+^, CD4^+^T: TCRβ^+^CD4^+^, CD8^+^T: TCRβ^+^CD8^+^, NK1.1^+^: FSC/NK1.1^+^. †Plasma B cells express CD93 as well. Data are represented as mean ± SD. One representative of two experiments is shown

Spleen leucocyte population changes were analysed during *T b brucei* AnTat1.1E infections. Table 1 shows the number of spleen, early B lineage (encompassing all CD93^+^ B cells), plasma B, follicular (Fo)B, marginal zone (MZ)B, CD4^+^ T, CD8^+^ T, and NK1.1^+^ cells, monocytes and neutrophils throughout infection (see supplemental Figure 1 for FACS gating strategy). A major influx of mature neutrophils (CD11b^+^Ly6G^+^Ly6C^Int^) is observed as early as 4 dpi (Table 1, Figure 1A, 1), and cell numbers remain elevated throughout infection (Figure 1B). Figure 1C displays the *T b brucei* AnTat 1.1E parasitemia profile.

**Table 1:** Immune cell populations in the spleen of *Trypanosoma brucei brucei* infected C57BL/6 mice

Cell type	Mean of cells per spleen (n = 3)												
	Days post‐infection (dpi)												
	0	4	5	6	7	8	9	10	14	17	21	24	28
Spleen^a^	1,00E + 8	1,85E + 8	2,34E + 8	3,43E + 8	3,25E + 8	2,44E + 8	2,05E + 8	2,16E + 8	1,39E + 8	1,83E + 8	1,43E + 8	1,63E + 8	1,25E + 8
Early B lineage	1,59E + 6	1,31E + 6	1,78E + 6	5,03E + 6	5,29E + 6	3,15E + 6	1,92E + 6	4,71E + 6	1,47E + 6	4,71E + 6	1,07E + 7	4,17E + 6	5,14E + 6
Plasma B	8,19E + 5	1,00E + 6	1,34E + 6	8,23E + 6	1,85E + 7	1,48E + 7	1,98E + 7	2,19E + 7	7,29E + 6	8,72E + 6	5,54E + 6	7,84E + 6	5,82E + 6
Follicular B	5,76E + 7	9,86E + 7	1,33E + 8	1,60E + 8	1,46E + 8	9,03E + 7	4,54E + 7	3,31E + 7	2,58E + 7	2,96E + 7	1,86E + 7	1,57E + 7	1,13E + 7
Marginal zone B	4,07E + 6	6,57E + 6	7,57E + 6	7,96E + 6	7,14E + 6	1,70E + 6	7,96E + 5	2,72E + 5	1,00E + 5	5,90E + 4	3,99E + 4	1,25E + 5	1,52E + 5
CD4^+^ T	1,64E + 7	1,69E + 7	2,13E + 7	3,20E + 7	3,25E + 7	1,89E + 7	1,05E + 7	8,34E + 6	9,23E + 6	1,49E + 7	1,57E + 7	1,78E + 7	1,44E + 7
CD8^+^ T	9,76E + 6	9,55E + 6	1,34E + 7	1,91E + 7	1,45E + 7	8,72E + 6	3,85E + 6	2,99E + 6	3,47E + 6	2,46E + 6	2,31E + 6	2,64E + 6	1,93E + 6
NK1.1^+^	4,79E + 6	3,00E + 6	1,55E + 6	2,49E + 6	2,43E + 6	2,25E + 6	1,13E + 6	1,54E + 6	1,47E + 6	1,98E + 6	1,27E + 6	1,63E + 6	1,67E + 6
Monocyte	1,00E + 6	5,85E + 6	3,65E + 6	1,14E + 7	1,21E + 7	1,16E + 7	1,05E + 7	8,07E + 6	7,62E + 6	5,67E + 6	5,11E + 6	8,90E + 6	6,35E + 6
Neutrophil	2,00E + 6	1,02E + 7	6,87E + 6	1,29E + 7	4,66E + 6	6,99E + 6	7,59E + 6	2,00E + 7	2,95E + 7	2,80E + 7	2,68E + 7	1,91E + 7	1,87E + 7

Note

Splenocytes of uninfected control mice and *T b brucei* AnTat1.1E infected mice (n = 3 mice per time point) were stained for surface markers and analysed using flow cytometry. Fold change in cell number of <0.25 (dark red), 0.25‐0.5 (medium red), 0.5‐0.8 (light red), 1.25‐2 (light green), 2‐9 (medium green), and >9 (dark green) are displayed. Data are represented as mean of at least three mice per group. Flow cytometry selection criteria are identical to those described in Figure 1.

a Spleen cells are referred to as the number of viable spleen cells, obtained using a haemocytometer and Trypan Blue staining, after performing red blood cell lysis.

Coinciding with the clearance of the first parasitemia peak (6 dpi), a 5‐fold increase in spleen neutrophil cells is observed (Figure 1D, Table 1). The neutrophil cell number remains high throughout the progressing infection, reaching a 15‐fold increase following the control of the third peak of infection. In contrast, while monocyte, plasma B, and early B lineage cells increase immediately following the first wave of infection, cell numbers drop again towards the end of infection, albeit not to baseline levels. Moreover, MZB, FoB, CD4^+^ T and CD8^+^ T cells reach peak numbers following the clearance of first peak of parasitemia. Thereafter, progressing infection results in sustained loss of these cells, except for CD4^+^ T cells, which only show a transient reduction following the second peak of infection (10 dpi). The coinciding significant increase in early B lineage and plasma B cells (previously reported 11, 12), could result from extramedullary B lymphopoiesis, and polyclonal B cell activation and/or differentiation of MZB into plasma B cells. In contrast, NK1.1^+^ cell numbers (NK and NKT cells) reduce immediately following the onset of infection and remain severely depleted thereafter. Collectively, our data show that following the control of both the first and second *T b brucei* AnTat1.1E parasitemia peaks, a cumulative increase of neutrophils coincides with the destruction of other mature spleen lymphocyte populations.

## 4 DISCUSSION

While analysing trypanosomosis‐induced anaemia in the past, we reported the early influx (4 dpi) of neutrophils in the spleen of infected mice, preceding the first peak of parasitemia.^5^ Here, we show that this cellular recruitment persists throughout the entire course of infection, reaching a 15‐fold increase upon the control of the third peak of infection. At the same time, MZB, FoB, NK1.1^+^ and CD8^+^ T cells are all depleted due to the ongoing infection. This persistent infection‐associated neutrophil accumulation is remarkable, as neutrophils are usually characterized as short‐lived cells associated with acute immune responses, dying within a limited time after performing their function.^19^ However, several recent studies have indicated that neutrophils are capable of executing more diverse functions, including the regulation of inflammatory responses, and acting as effectors of the adaptive immune system.^20^, ^21^ Since neutrophils play an important role in regulating immune response during parasite infections, the dynamic change of this cell population was addressed in an experimental *T b brucei* infection setup.

Following the observed persistent infection‐associated influx of spleen neutrophils during *T b brucei* AnTat1.1E infections, two questions can be put forward, that is what is the role of these cells with respect to the control of parasitemia, and secondly, could these cells contribute to the observed infection‐associated pathology?

Two possible scenarios can be suggested in which neutrophils would contribute to the regulation of parasitemia, dampening the parasitemia load while other immune cells are being depleted. Indeed, neutrophils could contribute to parasitemia control by (a) phagocytosis, (b) granular secretion of antibacterial compounds, (c) release of neutrophil extracellular traps (NETs), and (d) the induction of a hostile inflammatory environment.^22^, ^23^, ^24^, ^25^, ^26^ The latter, that is the combined action of neutrophil‐derived tumour necrosis factor (TNF) and NO, could aid the significantly weakened remaining antibody response in maintaining a certain parasitemia control lever during later stages of infection. In addition, neutrophils can stimulate the adaptive immune response, as they activate splenic B cells through the release of B‐cell‐stimulating factors. This can lead to (a) improved B cell survival, (b) IgM antibody secretion, (c) IgG and IgA isotype switching and (d) somatic hypermutation induction.^27^ Finally, neutrophils can positively regulate antigen‐specific T cell responses and can act as antigen‐presenting cells.^28^, ^29^ Collectively, these neutrophil effector functions could all contribute to parasitemia control by triggering both innate and adaptive defence responses. In contrast, neutrophils can play a role in the establishment and persistence of the parasite infection. A recent study revealed that the rapid recruitment of neutrophils to the dermal bite site of *T b brucei* infected tsetse flies, did contribute to higher systematic parasitemia levels during the onset of infection.^17^

With respect to the second question and the possible role of persistent spleen neutrophil accumulation as part of the infection‐associated immunopathology, it should be noted that a link between trypanosomosis‐associated B cell depletion and the activation of the NK‐perforin pathway has been suggested.^30^ Hence, since neutrophils can be an additional source of perforin, they could possibly contribute to B cell depletion during infection and aggravate the reported detrimental NK cell activity. In an ever‐accelerating cycle of immunopathology, spleen B cell destruction and architecture disruption could than further drive inflammation by enhancing the influx of IFN‐γ producing neutrophils, fueling the ongoing type I inflammatory immune response.

## CONFLICT OF INTEREST

The authors declare that the research was conducted in the absence of any commercial of financial relationships that could be construed as a potential conflict of interest.

## AUTHOR CONTRIBUTIONS

Research design: CD MR SM. Research acquisition: VD HTTP ID IJ. Data analysis and interpretation: VD ID HTTP IJ CD MR SM. Drafting of paper: VD. Revising of paper: CD MR SM.

## Data Availability

The data that support the findings of this study are available from the corresponding author upon reasonable request.
