# Supplementary figures and images for: Trypanosoma brucei brucei causes a rapid and persistent influx of neutrophils in the spleen of infected mice
Source: Parasite Immunol. 2019 Aug 21;41(10):e12664. doi: 10.1111/pim.12664 (PMC6771705; doi:10.1111/pim.12664)

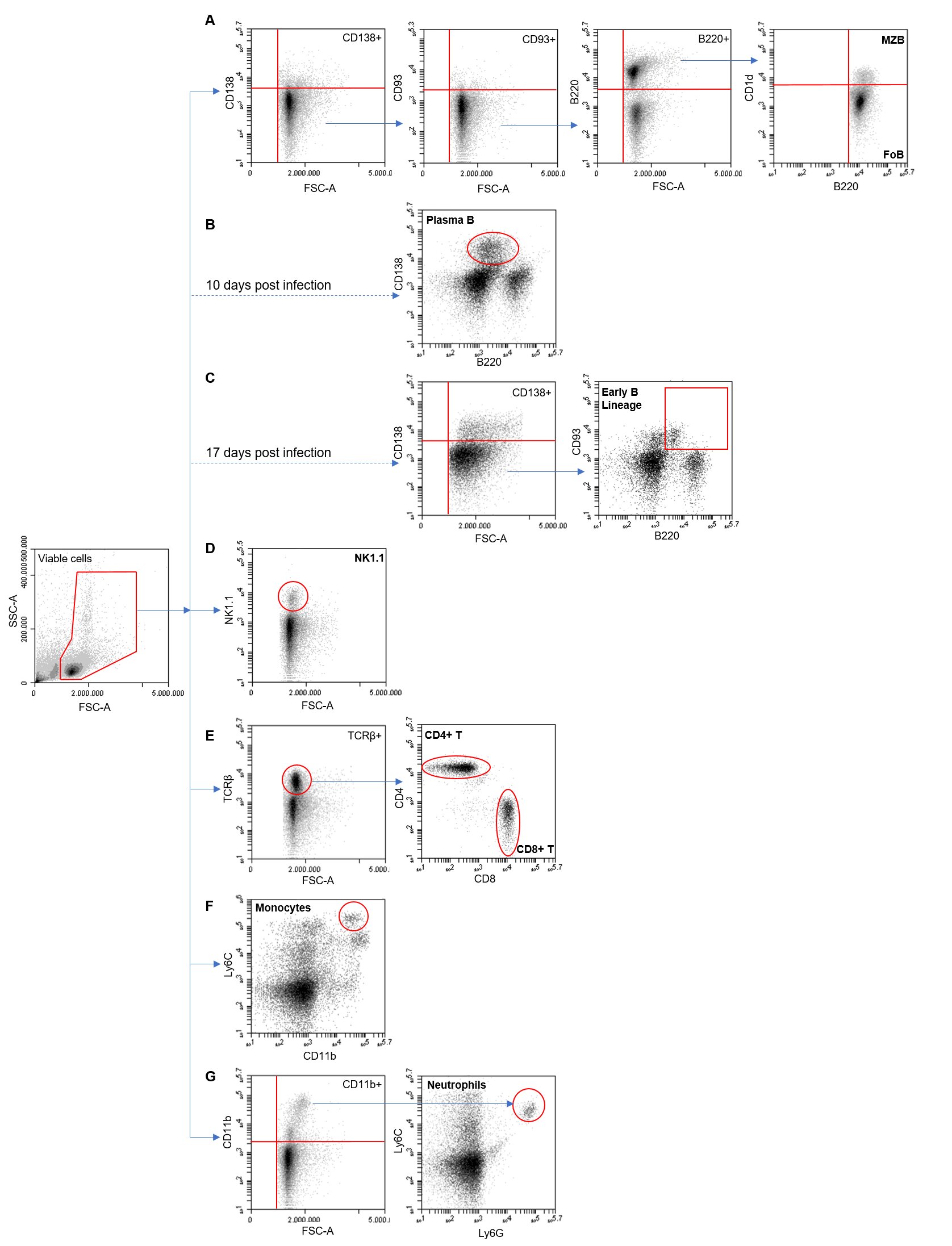

Supplement: Supplementary file 1 [file PIM-41-na-s001.jpg]
